# Disease progression patterns and risk factors associated with mortality in deceased patients with COVID‐19 in Hubei Province, China

**DOI:** 10.1002/iid3.343

**Published:** 2020-08-28

**Authors:** Liang Chen, Song Liu, Juncai Tian, Haisong Pan, Yu Liu, Jun Hu, Maoren Wang, Xuewen Hou

**Affiliations:** ^1^ Department of Radiology, Hubei Province Academy of Traditional Chinese Medicine Hubei Provincial Hospital of Traditional Chinese Medicine Wuhan China; ^2^ Department of Radiology, Yichang Central People's Hospital The First College of Clinical Medical Science Yichang China; ^3^ Department of Respiratory Medicine, The First People's Hospital of Ziyang West China Hospital Sichuan University‐Ziyang Hospital Ziyang China; ^4^ Department of Ophthalmology University Medical Center, Johannes Gutenberg University Mainz Mainz Germany; ^5^ Department of Internal Medicine, Campus Virchow Clinic, German Heart Center Berlin Charité University Medicine Berlin Germany

**Keywords:** COVID‐19, disease progression pattern, SARS‐CoV‐2, SOFA score

## Abstract

**Background:**

Detailed descriptions of the patterns of disease progression of deceased coronavirus disease 2019 (COVID‐19) patients have not been well explored.

**Objectives:**

This study sought to explore disease progression patterns and risk factors associated with mortality of deceased patients with COVID‐19.

**Materials and Methods:**

Epidemiological, clinical, laboratory, and imaging data (from 15 January to 26 March 2020) of laboratory‐confirmed COVID‐19 patients were collected retrospectively from two hospitals, Hubei province, China. Disease progression patterns of patients were analyzed based on laboratory data, radiological findings, and Sequential Organ Failure Assessment (SOFA) score. Risk factors associated with death were analyzed.

**Results:**

A total of 792 patients were enrolled in this study, of whom 68 died and 724 survived. Complications during hospitalization, such as sepsis, severe acute respiratory distress syndrome, acute cardiac injury, and acute kidney injury, were markedly more frequent in deceased patients than in surviving patients. Deceased patients presented progressive deterioration pattern in laboratory variables, chest computed tomography evaluation, and SOFA score, while surviving patients presented initial deterioration to peak level involvement followed by improvement pattern over time. Days 10 to 14 after illness onset was a critical stage of disease course. Older age, number of preexisting comorbidities ≥2, and SOFA score were independently associated with death for COVID‐19.

**Conclusions:**

Multiorgan dysfunction was common in deceased COVID‐19 patients. Deceased patients presented progressive deterioration pattern, while surviving patients presented a relatively stable pattern during disease progression. Older age, number of preexisting comorbidities ≥2, and SOFA score were independent risk factors for death for COVID‐19.

## Graphical abstract

Multiorgan dysfunction was common in deceased coronavirus disease 2019 (COVID‐19) patients. Deceased patients presented progressive deterioration pattern, while surviving patients presented a relatively stable pattern during disease progression. Older age, preexisting comorbidities, and Sequential Organ Failure Assessment score were independent risk factors for death for COVID‐19.

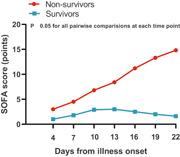

## 1 INTRODUCTION

A novel coronavirus infection‐related pneumonia was first reported in Wuhan, Hubei Province, China in December 2019.^1^, ^2^ Coronaviruses are enveloped RNA viruses that are distributed broadly in humans and other mammals, and cause diseases involving multiple systems.^3^ Similar to the two other coronavirus—severe acute respiratory syndrome coronavirus and Middle East respiratory syndrome coronavirus, the novel coronavirus, which has been named severe acute respiratory syndrome coronavirus 2 (SARS‐CoV‐2) by the International Committee on Taxonomy of Viruses on 11 February 2020,^4^ can also lead to severe acute respiratory distress syndrome (ARDS) in humans.^5^ This disease caused by SARS‐CoV‐2 has been renamed coronavirus disease 2019 (COVID‐19) by the World Health Organization (WHO) on 11 February 2020.^6^

Several previous studies^2^, ^5^, ^7^, ^8^ have reported the clinical features of patients with COVID‐19, including fever, cough, shock, ARDS, acute cardiac injury, and death. High‐revolution computed tomography (CT) is likely to become increasingly important for the diagnosis and management of COVID‐19. Chest CT findings of patients with COVID‐19 have also been reported.^9^, ^10^ In addition, the Sequential Organ Failure Assessment (SOFA) score has been proposed as a useful scoring tool to determine the level of organ dysfunction and mortality risk by the Sepsis‐3.0 version in 2016.^11^ However, patterns of disease progression of deceased patients with COVID‐19 have not been thoroughly described. Thus, the aim of this study was to investigate disease progression pattern of deceased patients with COVID‐19 by laboratory data, radiological findings, and SOFA score, as well as analyze risk factors for in‐hospital death, to improve overall understanding for COVID‐19.

## 2 METHODS

### 2.1 Study design and population

This study was a retrospective study conducted in two tertiary hospitals in Hubei Province: Yichang Central People's Hospital, Hubei Provincial Hospital of Traditional Chinese Medicine. Between 15 January 2020 and 26 March 2020, patients with confirmed SARS‐CoV‐2 infection according to the WHO technical guidance for human COVID‐2019 infection were enrolled.^12^ Patients who age less than 18 years, and died within the 7 days on admission were excluded.

Our institutional review boards approved this study, and that the study conforms to the Declaration of Helsinki; The requirement for informed consent was waived for emerging infectious diseases.

### 2.2 Data collection and analysis

The electronic medical records of patients with COVID‐19 were extracted and analyzed by the research team. Epidemiological history, clinical characteristics, laboratory data, radiological findings, treatments, and clinical outcomes were obtained. Subsequently, these data were reviewed by a trained team of physicians. The information assessed included epidemiological history, chronic medical history, symptoms at illness onset, laboratory data, chest CT examinations, treatment measures, and outcomes. The date of disease onset was defined as the day when the first symptoms were observed.

During patients hospitalization, CT scans were at the discretion of the treating clinicians as appropriate for clinical Scenario. CT images were reviewed by two radiologists (SL, LC, with 5‐10 years of clinical experience) who were blinded to the clinical data, and final decisions were reached by consensus. The distribution (central, peripheral, and mixed) and pattern (ground‐grass opacity [GGO], consolidation, crazy‐paving pattern, and mixed pattern) of lung abnormalities on CT were assessed and analyzed. The number of lung zones involved was recorded. The extent of lung abnormalities on CT was assessed by a semiquantitative scoring system.^13^ Each lung was classified into three lung regions: the upper lung zone (above the carina), the lower lung zone (below the inferior pulmonary vein), and the middle lung zone (between the upper and the lower lung zone). Each of the six lung zones was scored according to the percentage of lung involvement as follows: score 0, no involvement; score 1, less than 25% involvement; score 2, 25% to 50% involvement; score 3, 50% to 75% involvement; and score 4, more than 75% involvement. An overall lung score was calculated by summing the six lung zone scores, with values ranging from 0 to 24 for each patient.

SOFA scores (range: 0‐24 points) were calculated using physiological and laboratory variables, incorporating several parameters: respiration (PaO_2_/FiO_2_), coagulation (platelets count), liver (bilirubin), cardiovascular (hypotension), central nervous system (Glasgow Coma Score), and renal (creatinine) as previously described.^14^

### 2.3 Definitions

ARDS was defined according to the Berlin Definition.^15^ Sepsis and septic shock were defined according to International Guidelines for Management of Sepsis and Septic Shock: 2016.^16^ Acute cardiac injury was diagnosed if serum levels of cardiac biomarkers (eg, high‐sensitivity cardiac troponin I) were above the 99th percentile upper reference limit, or if new abnormalities were demonstrated in electrocardiography and echocardiography. Coagulopathy was defined as a 3‐second extension of prothrombin time (PT) or a 5‐second extension of activated partial thromboplastin time. Acute kidney injury was diagnosed according to the Kidney Disease: Improving Global Outcomes definition.^17^

Regarding the definitions of chest CT image patterns, the GGO pattern was defined as hazy increased opacity of lung, with preservation of bronchial and vascular margins on CT scans. Consolidation pattern is defined as a homogeneous increase in pulmonary parenchymal attenuation that obscures the margins of vessels and airway walls on radiographs and CT scans, an air bronchogram may be present. Crazy‐paving pattern is defined as thickened interlobular septa and intralobular lines superimposed on a background of ground‐glass opacity, resembling irregularly shaped paving stones according to the Fleischner Society glossary of terms for thoracic imaging.^18^ A mixed pattern was defined as a combination of GGO and consolidation.

Evaluation of disease progression pattern over time of COVID‐19

### 2.4 Dynamic change in laboratory parameters

Laboratory variables including high‐sensitivity lymphocyte count, cardiac troponin I (hsTnI), D‐dimer, lactate dehydrogenase (LDH), serum creatinine, and blood urea nitrogen (BUN) obtained during hospitalization (at a 3 days time interval from day 4 after illness onset) were selected to describe dynamics of disease progression.

### 2.5 Dynamic change in chest CT findings

The serial chest CT score and image patterns (GGO, consolidation, crazy‐paving pattern, and mixed pattern) of lung lesions in different periods (days 1‐5, 6‐9, 10‐14, 15‐20, 21‐28 after illness onset, respectively) were analyzed to describe dynamics of disease progression. Only patients who underwent at least three times of CT scans in the same patients in the aforementioned time windows could be selected.

### 2.6 Dynamic change in SOFA score

SOFA score calculated during hospitalization (at a 3 days time interval from day 4 after illness onset) was used to describe the dynamics of disease progression.

### 2.7 Statistical analysis

Data for categorical variables are expressed as frequency rates and percentages and were compared using the *χ* ^2^ test. Continuous variables are described using the mean, median, and interquartile range (IQR), and differences between groups were compared using the Student *t* test (normally distributed data) or the Mann‐Whitney *U* test (nonnormally distributed data). Univariate and multivariate logistic regression models were constructed to determine variables that were associated with in‐hospital death. First, variables on admission were included to conduct the univariate analysis; Second, variables with *P* < .10 were enrolled in the multivariate model with the backward stepwise method. Differences were considered significant at *P* < .05 with a two‐tailed test. The analyses were performed with statistical packages (SPSS 26.0, Chicago; GraphPad Prism 8.2, San Diego).

## 3 RESULTS

### 3.1 Population characteristics

A total of 792 adult patients with laboratory‐confirmed SARS‐CoV‐2 infection and met study criteria were enrolled in this study, including 68 patients who died and 724 who surviving. As shown in Table 1, 92 (12%) patients had traveled to Wuhan within 2 weeks of the onset of symptoms. The median age of deceased patients was 70 (IQR: 68.0‐77.5) years, which was markedly older than that of surviving patients (52 [34.5‐65.0] years); underlying diseases (diabetes mellitus, hypertension, and chronic kidney disease) were much more frequent in deceased patients. The most common symptoms at illness onset were fever and cough, followed by myalgia or fatigue in both deceased patients and surviving patients. Deceased patients demonstrated a significantly higher SOFA score than that in surviving patients. The incidence of complications during hospitalization, such as sepsis, respiratory failure, ARDS, heart failure, shock, acute cardiac injury, and acute kidney injury, was notably more frequent in deceased patients than in surviving patients (Table 1). In the intensive care unit, most deceased patients received noninvasive and/or invasive mechanical ventilation therapy. The median time from illness onset to discharge was 22.5 (IQR: 19.0‐25.0) days, whereas the median time to death was 19.0 days (16.5‐24.0) (Table 1).

**Table 1:** Demographics and clinical characteristics of the patients

Variable	All (n = 792)	Survivors (n = 724)	Nonsurvivors (n = 68)	*P* value
Age, y	55.0 (36.0‐68.0)	52.0 (34.5‐65.0)	70.0 (68.0‐77.5)	<.001
Men	432 (55%)	398 (55%)	34 (50%)	.431
Wuhan exposure	92 (12%)	72 (10%)	20 (29%)	<.001
Comorbidities				
Hypertension	215 (27%)	181 (25%)	34 (50%)	<.001
Diabetes	142 (18%)	116 (16%)	26 (38%)	<.001
COPD	19 (2%)	14 (2%)	5 (7%)	.005
Chronic kidney disease	26 (3%)	21 (3%)	5 (7%)	.049
Initial symptoms				
Fever	623 (79%)	564 (78%)	59 (87%)	.088
Cough	588 (74%)	536 (74%)	52 (76%)	.660
Expectoration	146 (18%)	130 (18%)	16 (24%)	.257
Myalgia or fatigue	254 (32%)	224 (31%)	30 (44%)	.026
Shortness of breath	49 (6%)	43 (6%)	6 (9%)	.345
Diarrhea	56 (7%)	51 (7%)	5 (7%)	.924
Time from illness onset to first hospital admission, d	4.0 (2.7‐6.0)	4.0 (2.0‐6.1)	4.0 (3.0‐5.0)	.909
SOFA score (points)	1.5 (1.0‐2.5)	1.0 (1.0‐2.0)	3.0 (2.0‐4.0)	<.001
Complications				
ARDS	104 (13%)	43 (6%)	61 (90%)	<.001
Respiratory failure	252 (32%)	188 (26%)	64 (94%)	<.001
Sepsis	321 (41%)	253 (35%)	68 (100%)	<.001
Sepsis shock	39 (5%)	0 (0)	39 (57%)	<.001
Secondary infection	35 (4%)	7 (1%)	28 (41%)	<.001
Acute cardiac injury	40 (5%)	7 (1%)	33 (49%)	<.001
Acute kidney injury	46 (6%)	14 (2%)	32 (47%)	<.001
Treatment				
Antiviral therapy	654 (83%)	623 (86%)	21 (31%)	<.001
Corticosteroids	143 (18%)	116 (16%)	27 (40%)	<.001
Antibiotic therapy	715 (90%)	651 (90%)	64 (94%)	.264
High‐flow oxygen therapy	90 (11%)	51 (7%)	39 (57%)	<.001
ICU admission	94 (12%)	44 (6%)	50 (74%)	<.001
Noninvasive ventilation	44 (6%)	14 (2%)	30 (44%)	<.001
Invasive mechanical ventilation	47 (6%)	7 (1%)	40 (59%)	<.001
Renal replacement therapy	11 (1%)	0 (0)	11 (16%)	<.001
Days from illness onset to death or discharge death or discharge, d	21.0 (18.0‐24.0)	22.5 (19.0‐25.0)	19.0 (16.5‐24.0)	.094

*Note:* Data are expressed as the median (interquartile range) or n (%).

Abbreviations: ARDS, acute respiratory distress syndrome; COPD, chronic obstructive pulmonary disease; ICU, intensive care unit; SOFA, Sequential Organ Failure Assessment.

### 3.2 Baseline laboratory findings

As shown in Table 2, there were significant differences in baseline laboratory findings between patients who died and those who survive. Compared with surviving patients, deceased patients displayed higher levels of D‐dimer, procalcitonin, high‐sensitivity troponin I (hsTnI), creatine kinase, alanine aminotransferase, aspartate aminotransferase, total bilirubin, serum creatinine, blood BUN, LDH, and erythrocyte sedimentation rate, as well as higher baseline white blood cell count and longer PT. In addition, deceased patients showed a markedly lower lymphocyte count.

**Table 2:** Baseline laboratory data and chest CT findings of patients infected with COVID‐19

Variable		All (n = 792)	Survivors (n = 724)	Nonsurvivors(n = 68)	*P* value
Laboratory data	Normal range				
White blood cell count (×10^9^/L)	4‐10	5.5 (4.6‐7.8)	4.9 (4.1‐7.1)	8.4 (5.6‐10.3)	.001
Lymphocyte count (×10^9^/L)	1.1‐3.2	0.9 (0.6‐1.2)	0.9 (0.6‐1.3)	0.6 (0.4‐0.9)	<.001
Platelets count (×10^9^/L)	125‐350	215 (160‐258)	225 (172‐270)	170 (110‐230)	<.001
C‐reactive protein, mg/L	<10	10.5 (5.4‐28.0)	8.4 (4.5‐16.5)	56.9 (28.2‐79.4)	<.001
Procalcitonin, ng/mL	<0.05	0.06 (0.03‐0.14)	0.05 (0.03‐0.09)	0.15 (0.09‐0.47)	<.001
ESR, mm/h	<20	21.0 (10.0‐42.0)	18.5 (9.5‐36.2)	72.0 (28.0‐92.5)	<.001
Prothrombin time, s	11.5‐14.5	14.2 (13.6‐14.6)	14.0 (13.4‐15.0)	14.8 (14.0‐16.8)	.008
D‐dimer, mg/L	≤500	245 (181‐425)	202 (154‐308)	422 (271‐846)	<.001
hsTnI, pg/mL	<28	4.4 (2.1‐12.6)	3.5 (1.4‐7.9)	9.9 (6.6‐15.2)	<.001
Creatine kinase, U/L	40‐200	84 (62‐185)	78 (57‐157)	162 (108‐281)	.001
LDH, U/L	135‐225	276 (192‐326)	218 (178‐290)	386 (317‐529)	<.001
ALT, U/L	<40	28.0 (20.0‐43.0)	26.0 (19.0‐41.0)	32.0 (24.0‐49.0)	.003
AST, U/L	<40	29.0 (26.0‐44.0)	27.0 (23.5‐39.0)	40.0 (30.0‐56.0)	<.001
Total bilirubin, μmol/L	≤26	10.2 (9.4‐13.9)	9.8 (8.9‐13.1)	11.4 (9.2‐16.2)	.018
Serum creatinine, μmol/L	59‐104	69.0 (58.0‐88.0)	66.0 (52.5‐82.0)	88.5 (66.0‐144)	.001
Blood urea nitrogen, mmol/L	3.6‐9.5	5.0 (3.4‐6.8)	4.2 (3.0‐5.6)	6.2 (5.4‐9.3)	<.001
Chest CT findings					
Involved lung					
Bilateral		577 (73%)	521 (72%)	56 (82%)	.065
Number of lobes affected					
≤3		433 (55%)	420 (58%)	13 (19%)	<.001
>3		359 (45%)	304 (42%)	55 (81%)	<.001
Distribution of opacification					
Central		35 (5%)	29 (4%)	6 (9%)	.065
Peripheral		548 (69%)	514 (71%)	34 (50%)	<.001
Both central and peripheral		209 (26%)	181 (25%)	28 (41%)	.004
Patterns of opacification					
GGO		691 (87%)	637 (88%)	54 (79%)	<.001
Consolidation		158 (20%)	138 (19%)	20 (29%)	.041
Crazy‐paving pattern		140 (18%)	116 (16%)	24 (35%)	<.001
Mixed pattern		70 (9%)	58 (8%)	12 (18%)	.008
Other findings					
Lymphadenopathy		11 (2%)	7 (1%)	4 (6%)	.001
Pleural effusion		10 (1%)	7 (1%)	3 (4%)	.015
Initial chest CT score		3.5 (3.0‐4.5)	3.0 (2.5‐4.0)	6.0 (4.5‐8.0)	<.001

*Note:* Data are expressed as median (interquartile range) or n (%).

Abbreviations: ALT, alanine aminotransferase; AST, aspartate aminotransferase; CT, computed tomography; COVID‐19, coronavirus disease 2019; ESR, erythrocyte sedimentation rate; GGO, ground‐grass opacity; hsTnI, high‐sensitivity troponin I; LDH, lactate dehydrogenase.

### 3.3 Initial CT characteristics

Lung abnormalities on initial CT are shown in Table 2. A total of 56 (82%) deceased patients and 521 (72%) surviving patients had bilateral involvement on chest CT scans. Deceased patients showed a greater number of lung zones affected more than 4 than surviving patients. Typical chest CT findings of COVID‐19 pneumonia included predominantly peripheral GGO, consolidation, crazy‐paving pattern (GGO with interstitial thickening), and mixed pattern in both deceased and surviving patients. However, deceased patients were more likely to have consolidation, crazy‐paving pattern, and mixed pattern of the lungs on admission. Less frequent findings included lymphadenopathy and pleural effusion. In addition, deceased patients had a higher CT score than surviving patients on admission.

### 3.4 Evaluation of pattern of disease progression

#### 3.4.1 Patterns of dynamic change in laboratory parameters in deceased and surviving COVID‐19 patients

**Figure 1:**
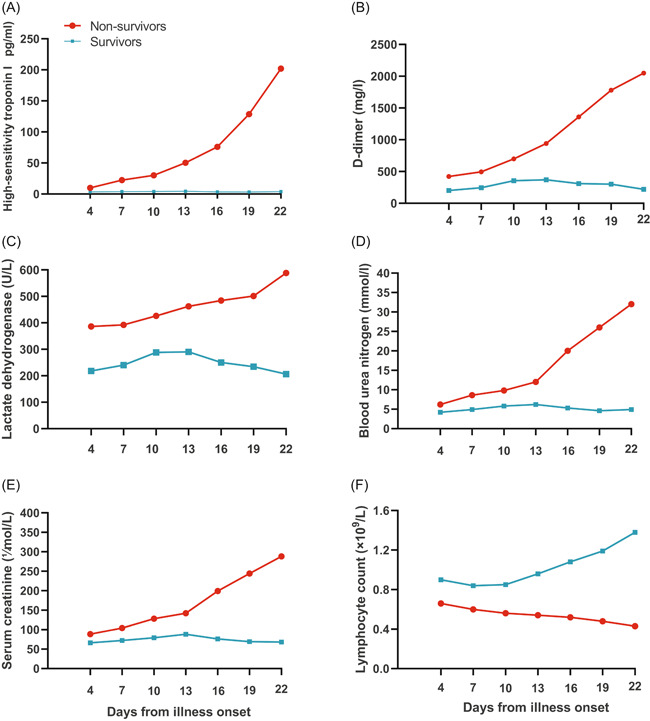
Pattern of dynamic changes in laboratory markers after disease onset in patients with COVID‐19. The graph shows dynamic changes in high‐sensitivity cardiac troponin I (A), d‐dimer (B), lactate dehydrogenase (C), blood urea nitrogen (D), serum creatinine (E), and lymphocytes (F) between patients who nonsurvivors (progressive deterioration pattern) and survivors (initial deterioration to peak level involvement followed by improvement pattern). COVID‐19, coronavirus disease 2019

The dynamic changes in laboratory parameters, including hematological and biochemical parameters, were tracked from illness onset (Figure 1). There were significant statistical differences in the dynamic changes in laboratory parameters between deceased and surviving patients. Most patients in the study had significant lymphopenia, and deceased patients developed more severe lymphopenia over time. The levels of high‐sensitivity cardiac troponin I, D‐dimer, LDH, BUN, and creatinine were higher in deceased patients than that in surviving patients during disease progression. Overall, deceased patients showed a progressive deterioration pattern of laboratory markers, and relatively more obvious deterioration occurred on 10 to 13 days after illness onset, while surviving patients showed a relatively stable pattern (initial deterioration to the peak level, followed by improvement) over time.

### 3.5 Pattern of dynamic change in CT score in deceased and surviving COVID‐19 patients

**Figure 2:**
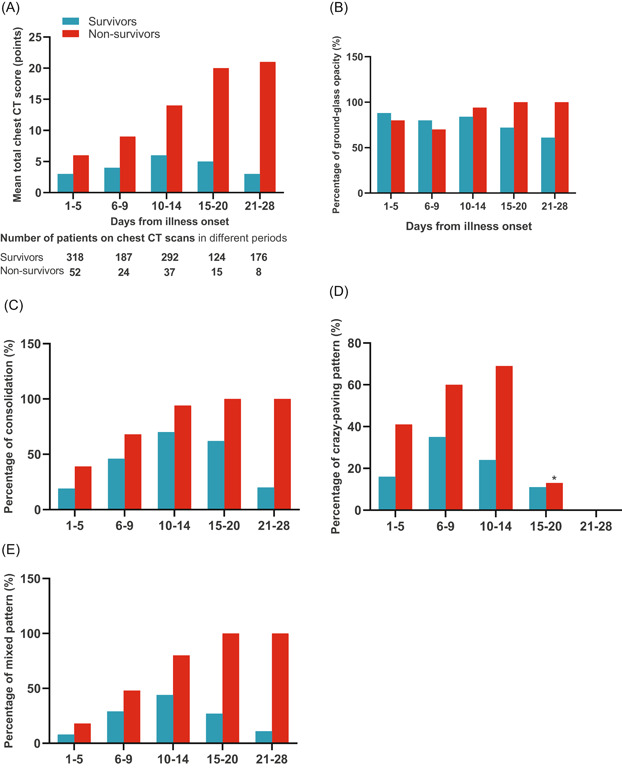
Pattern of dynamic changes in the total CT score and CT image pattern of lung lesions from disease onset in patients with COVID‐19. The graph shows dynamic changes in mean total CT score (A), percentage of consolidation (C), and percentage of mixed pattern (E) between nonsurvivors (progressive deterioration pattern) and survivors (initial deterioration to peak level involvement followed by improvement pattern). Percentage of ground‐grass opacity (B) is persistent more than 50% both in nonsurvivors and survivors within 28 days from illness onset, while the percentage of crazy‐paving pattern (D) is persistently increased until days 10 to 13, followed by decreased and no observed on days 21 to 28 both in nonsurvivors and survivors. COVID‐19, coronavirus disease 2019; CT, computed tomography

**Figure 3:**
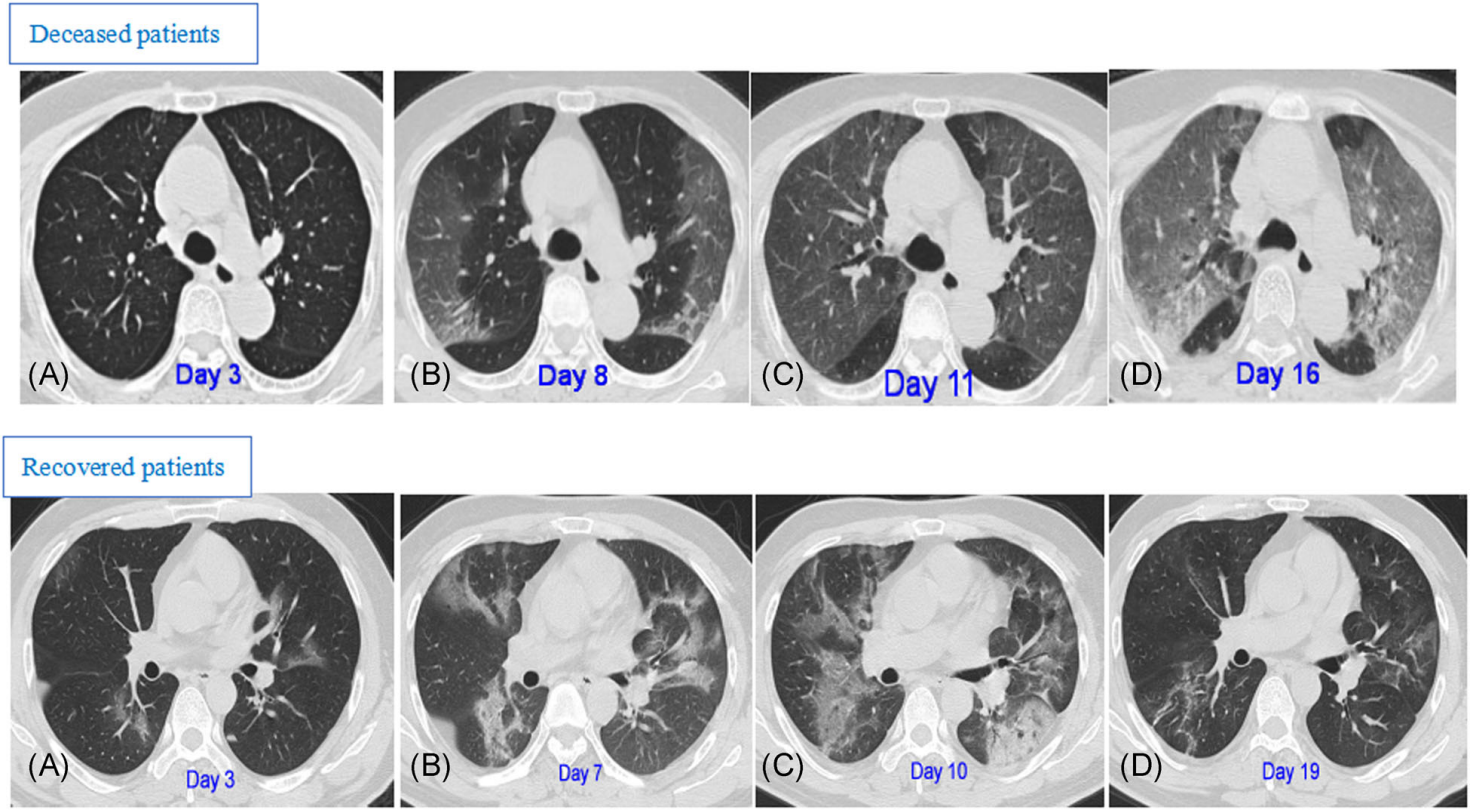
CT pattern of progression (upper panel A‐D) in an 83‐year‐old female deceased patient with fever and shortness of breath for 3 days. A, Initial CT (3 days from onset) only shows interlobular septal thickening in both lobes. B, Follow‐up CT obtained 5 days later shows multiple bilateral ground‐glass opacities (GGOs). C, Subsequent follow‐up CT obtained after another 3 days shows bilateral progressive CT progression. D, Final CT obtained after another 16 days shows “white lung” in both lungs, and the patient died of respiratory failure after 2 days. CT pattern of progression (lower panel A‐D) in a 48‐year‐old male recovered patient who presented with fever and cough for 3 days. A, Initial CT (3 days from onset): multiple irregular GGOs distributed bilaterally are shown. B, On day 7, there was a diffuse enlargement of GGO with partial consolidation and increased density. C, Day 10, further deteriorated GGO, which reached a peak level, with consolidation and air bronchogram. D, On day 19, the obvious resolution of GGO with fibrous stripes was observed. CT, computed tomography

The dynamic changes in chest CT score and the percentages of image patterns were analyzed and tracked from illness onset (Figures 2 and 3). Deceased patients showed a progressive deterioration pattern in chest CT score, percentage of consolidation, and percentage of mixed pattern with disease progression, while surviving patients showed initial deterioration to the peak level followed by improvement. Percentage of GGO was persistent more than 50% both in deceased patients and surviving patients within 28 days from illness onset, while the percentage of crazy‐paving pattern was persistently increased until days 10 to 14, followed by decreased and no observed on days 21 to 28 both in deceased patients and surviving patients.

### 3.6 Pattern of dynamic change in SOFA score in deceased and surviving COVID‐19 patients

**Figure 4:**
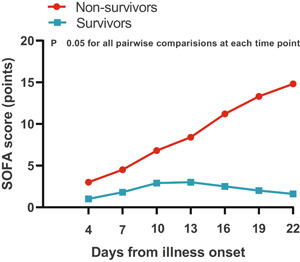
Dynamic changes in the SOFA score from disease onset in patients with COVID‐19. The graph shows dynamic changes in SOFA score between patients who nonsurvivors (progressive deterioration pattern) and survivors (initial deterioration to peak level involvement followed by improvement pattern). COVID‐19, coronavirus disease 2019; SOFA, Sequential Organ Failure Assessment

The dynamic change in the SOFA score was analyzed and tracked from illness onset (Figure 4). Similar to the overall change trend in laboratory parameters, there was a significant statistical difference in the dynamic change in SOFA score between deceased and surviving patients. Deceased patients showed a progressive deterioration pattern in SOFA score with disease progression, a more marked increased in SOFA score occurred after day 13 from illness onset, suggesting a sudden exacerbation of multiorgan dysfunction, most patients died during days 16 to 26, while surviving patients showed initial deterioration to the peak level followed by improvement.

### 3.7 Risk factors for death for COVID‐19

As shown in Table 3, multivariate analysis demonstrated that age (odds ratio [OR]: 1.05; 95% confidence interval [CI]: 1.01‐1.11; *P* < .001], number of preexisting comorbidities ≥2 (OR: 6.68; 95% CI: 2.14‐ 29.43; *P* = .002), and SOFA score (OR: 5.23; 95% CI: 1.98‐10.79; *P* < .001) were independently associated with death.

**Table 3:** Multivariate analysis for risk factors of mortality in patients with COVID‐19 using logistic regression model

Variable	OR (95% CI)	*P* value
Age	1.05 (1.01‐1.11)	.016
Number of preexisting comorbidities ≥2	6.68 (2.14‐29.44)	.002
SOFA score	5.23 (1.98‐10.79)	<.001

Abbreviations: CI, confidence interval; COVID‐19, coronavirus disease 2019; OR, odds ratio; SOFA, Sequential Organ Failure Assessment.

## 4 DISCUSSION

This was a retrospective study on the patterns of disease progression in laboratory tests, chest CT scans, and SOFA score in deceased and surviving patients with COVID‐19, as well as risk factors for death for COVID‐19. The current study found that (a) deceased patients were markedly older and had a higher prevalence of comorbidities, displayed more obvious abnormalities in laboratory parameters, and more severe lung infiltration, as well as higher SOFA score than in surviving patients on admission. (b) Overall, deceased patients presented progressive deterioration pattern in laboratory variables, chest CT evaluation, and SOFA score, while surviving patients presented initial deterioration to peak level involvement followed by improvement pattern during disease progression. Days 10 to 14 after illness onset was a critical stage of disease course. (c) Older age, number of preexisting comorbidities ≥2, and SOFA score were independent risk factors for death for COVID‐19.

The initial symptoms did not differ significantly between deceased patients and surviving patients. Deceased subjects were markedly older and had a higher prevalence of comorbidities than surviving subjects, suggesting that older age and comorbidities may be risk factors for COVID‐19‐related mortality. Age and comorbidities were independently associated with death in patients with SARS and MERS, which was reported.^19^, ^20^ Not surprisingly, several previous studies have reported older age and preexisting conditions were associated with mortality in COVID‐19 patients.^21^, ^22^, ^23^ In fact, it is not surprising that age and preexisting illness increases the risk of death or a complicated course for many diseases. Similarly, the current study also confirmed that older age and preexisting comorbidities were independent predictors of in‐hospital death for COVID‐19.

Significant differences in abnormalities in baseline and follow‐up laboratory parameters between the deceased patients and the surviving patients were found. Lymphopenia was more common in deceased patients. Furthermore, deceased patients were more likely to develop multiple organ dysfunction than surviving patients. Multiple organ involvement, including the kidney, liver, and gastrointestinal tract, was reported in patients with SARS.^24^ In our study, most deceased patients had multiorgan abnormalities of the cardiac, liver, kidney, and immune systems during treatment, which is consistent with previous studies.^25^, ^26^ Deceased patients had more lung zones affected (>4) and a higher incidence of consolidation of opacities than recovered patients on initial and follow‐up CT. Severe lung infiltration was more likely to cause poor outcomes. According to a study published previously,^27^ massive formation of transparent membranes in the alveolar cavity was found in deceased COVID‐19 patients. This results in a decrease in pulmonary compliance and an imbalance in ventilation and blood flow. The clinical features included massive consolidation visible on chest imaging, breathing difficulty, respiratory distress, and obstinate hypoxemia.

Regarding disease progression patterns, we found that deceased patients showed progressive deterioration of laboratory markers during disease course, while surviving patients presented a relatively stable pattern over time, which was consistent with the previous report.^21^ On chest CT, deceased patients showed a progressive deterioration pattern in CT score, percentage of consolidation, and percentage of mixed pattern, indicating persistent deterioration of pneumonia, while recovered patients showed initial deterioration to the peak level followed by improvement over time. The SOFA score is a good diagnostic marker for sepsis and septic shock, and is a useful scoring tool to determine level of organ dysfunction and mortality risk.^11^, ^28^ In our study, deceased patients showed a progressive deterioration pattern in SOFA score, while surviving patients showed initial deterioration to the peak level followed by improvement over time. Significant higher SOFA after 14‐day illness onset suggesting occurrence of multiple organ dysfunction. Furthermore, our study proved that SOFA score is independently associated with mortality, which was consistent with the previous report.^21^

Overall, these patterns presented indicate that the disease progression of COVID‐19 reached its peak level on 10 to 14 days after illness onset in surviving patients and that patients with progressive increases in the level of laboratory markers, CT score, and SOFA score after 14 days are more likely to have a poor prognosis. Fourteen days may be a watershed for determining disease outcomes. So far, there is no specific effective drug for COVID‐19 treatment, but earlier supportive treatments might alter disease progression. Description of patterns of disease progression of COVID‐19 may provide an overall understanding of the course of this acute severe infectious disease.

This study has several limitations. First, this is a retrospective study, making it difficult to avoid selection bias. Second, we did not explore dynamic changes in the viral load, and this may be warranted in future studies.

In summary, multiorgan dysfunction was common in deceased patients with COVID‐19. Deceased patients presented progressive deterioration pattern in laboratory variables, chest CT evaluation, and SOFA score, while surviving patients presented initial deterioration to peak level involvement followed by improvement pattern during disease progression. Older age, number of preexisting comorbidities ≥2, and SOFA score were independent risk factors for death for COVID‐19.

## CONFLICT OF INTERESTS

The authors declare that there are no conflict of interests.

## AUTHOR CONTRIBUTIONS

LC, SL, JT, HP, YL, and JH analyzed and interpreted the data. LC, MW, and XH performed the statistical analysis and wrote the manuscript. All authors read and approved the final manuscript.

## Data Availability

After publication, data are available upon reasonable request. A proposal with a detailed description of study objectives and statistical analysis plan will be needed for evaluation of the reasonability of requests. Additional materials might also be required during the process of evaluation. Deidentified participant data will be provided after approval from the corresponding author and Yichang Central People's Hospital, Hubei Provincial Hospital of Traditional Chinese Medicine.
